# The *Arabidopsis* USL1 controls multiple aspects of development by affecting late endosome morphology

**DOI:** 10.1111/nph.15249

**Published:** 2018-06-13

**Authors:** Rongrong Yuan, Jingqiu Lan, Yuxing Fang, Hao Yu, Jinzhe Zhang, Jiaying Huang, Genji Qin

**Affiliations:** ^1^ State Key Laboratory of Protein and Plant Gene Research School of Life Sciences School of Advanced Agricultural Sciences Peking University Beijing 100871 China; ^2^ The Peking‐Tsinghua Center for Life Sciences Academy for Advanced Interdisciplinary Studies Peking University Beijing 100871 China

**Keywords:** *Arabidopsis thaliana*, AtVPS30, AtVPS34 complex, auxin transport, endocytic trafficking, late endosome, USL1, VPS29

## Abstract

- The polar transport of auxin controls many aspects of plant development. However, the molecular mechanisms underlying auxin tranport regulation remain to be further elucidated.
- We identified a mutant named as *usl1* (***u**nflattened and **s**mall **l**eaves*) in a genetic screen in *Arabidopsis thaliana*. The *usl1* displayed multiple aspects of developmental defects in leaves, embryogenesis, cotyledons, silique phyllotaxy and lateral roots in addition to abnormal leaves. *USL1* encodes a protein orthologous to the yeast vacuolar protein sorting (Vps) 38p and human UV RADIATION RESISTANCE‐ASSOCIATED GENE (UVRAG). Cell biology, Co‐IP/MS and yeast two‐hybrid were used to identify the function of USL1.
- USL1 colocalizes at the subcellular level with VPS29, a key factor of the retromer complex that controls auxin transport. The morphology of the VPS29‐associated late endosomes (LE) is altered from small dots in the wild‐type to aberrant enlarged circles in the *usl1* mutants. The *usl1* mutant synergistically interacts with *vps29*. We also found that USL1 forms a complex with AtVPS30 and AtVPS34.
- We propose that USL1 controls multiple aspects of plant development by affecting late endosome morphology and by regulating the PIN1 polarity. Our findings provide a new layer of the understanding on the mechanisms of plant development regulation.

## Introduction

The phytohormone auxin plays essential roles in control of many aspects of plant development from embryogenesis to postembryonic development as a morphogen by specific distribution through its biosynthesis, conjugation, metabolism and polar transport (Friml, 2003; Qin *et al*., 2005; Teale *et al*., 2006; Cheng *et al*., 2007; Baylis *et al*., 2013; Kazan, 2013; Bar & Ori, 2014; Kasprzewska *et al*., 2015; Tang *et al*., 2016). At the cellular level, the asymmetric subcellular distribution of the PIN‐FORMED (PIN) auxin transport family proteins generate local auxin gradients key for plant development by driving auxin to be transported from cell to cell in a polar manner (Gälweiler *et al*., 1998; Müller *et al*., 1998; Tanaka *et al*., 2006). Therefore, the changes of the PIN family proteins in subcellular organelles indirectly regulate plant development by affecting auxin distribution.

PIN proteins undergo constitutive clathrin‐mediated endocytosis to subsequently be recycled to different polar domains (Geldner *et al*., 2003; Dhonukshe *et al*., 2007; Tanaka *et al*., 2009; Feraru *et al*., 2012; Naramoto *et al*., 2014b) or to be delivered to vacuoles for degradation (Kleine‐Vehn *et al*., 2008; Spitzer *et al*., 2009; Baster *et al*., 2013). Briefly, PIN proteins on the plasma membrane (PM) can be internalized by membrane invagination to form clathrin‐coated vesicles (CCVs). CCVs first reach the trans‐Golgi network/early endosomes (TGN/EE). From there, the PIN proteins are sorted back to the PM for reuse via recycling endosomes or delivered to vacuoles for degradation through late endosomes/multivesicular bodies/prevacuolar compartments (LE/MVB/PVC) (Grunewald & Friml, 2010). GNOM and ESCRT (endosomal sorting complex required for transport) complex has been identified to be essential for the recycling or degradation of the PIN proteins, respectively. *GNOM* encodes a guanine nucleotide exchange factor for ADP‐ribosylation factors (ARF‐GEF) (Shevell *et al*., 1994; Busch *et al*., 1996; Steinmann *et al*., 1999). The active GTP‐bound ARFs are important for the formation, targeting and fusion of vesicles in endocytic trafficking because of their tight binding to the membrane in eukaryotic organisms (Donaldson & Jackson, 2000; Jürgens & Geldner, 2002). GNOM can activate ARFs by changing a GDP in the inactive ARFs to be a new GTP in control of the PIN1 endocytic cycling (Geldner *et al*., 2003). The disruption of GNOM causes abnormal PIN1 polarity and severe defects in plant embryogenesis. The ESCRT complex includes five subcomplexes including ESCRT‐0, ESCRT‐I, ESCRT‐II, ESCRT‐III and VPS4 that coordinate to sort ubiquitinated PM proteins into intraluminal vesicles (ILV) inside the lumen of the LE/MVB/PVC for their final degradation in vacuoles (Piper & Katzmann, 2007; Otegui & Spitzer, 2008; Reyes *et al*., 2011). Several reports have demonstrated that some subunits of the plant ESCRT complex play critical roles in mediating the degradation of the PIN proteins in the vacuoles using the endosomal sorting pathway (Spitzer *et al*., 2009; Gao *et al*., 2014; Wang *et al*., 2017). In contrast to the function of the ESCRT complex, the retromer complex retrieves the PIN proteins from the LE/MVB/PVC to return them to the TGN/EE via the retrograde pathway to avoid PIN degradation in the lytic vacuoles (Kleine‐Vehn *et al*., 2008).

The retromer is a conserved complex localized to the cytosolic face of the endosomes and is highly important for the intracellular sorting of PM transporters and receptors in eukaryotes (Seaman, 2005; Bonifacino & Rojas, 2006; Bonifacino & Hurley, 2008; Zelazny *et al*., 2013). The multimeric retromer is composed of two subcomplexes. One is the core subcomplex containing the trimer of VPS26, VPS29 and VPS35 that is proposed to bind directly to the cytosolic tail of cargo proteins. The other consists of the dimer of the sorting nexin proteins (SNXs). The *Arabidopsis VPS29* is a single copy gene, and the *vps29* loss‐of‐function mutant displays severe auxin‐related phenotypes including defective embryogenesis, abnormal cotyledons, fewer lateral roots, dwarfism and agravitropism (Shimada *et al*., 2006; Jaillais *et al*., 2007). Genetic and molecular evidence indicates that VPS29 is required for the proper subcellular trafficking of the PIN proteins by maintaining the morphology of the LE/MVB/PVC (Jaillais *et al*., 2007). The *Arabidopsis* genome contains three VPS35 homologs designated as VPS35a, VPS35b and VPS35c. VPS35a plays the predominant role in PIN endocytic trafficking. Although the *vps35a* mutant exhibits no obvious phenotypes such as those observed in *vps29*, the altered morphology of the LE/MVB/PVC and the abnormal intracellular localization of the PIN proteins in *vps35a* are similar to those in *vps29* (Nodzyński *et al*., 2013). In addition, the triple mutant *vps35a vps35b vps35c* is embryo‐lethal, indicating that the core retromer complex is pivotal for plant development (Yamazaki *et al*., 2008). VPS26 has two copies denominated VPS26a and VPS26b in *Arabidopsis*. The double mutant *vps26a vps26b* displays defects in seedling development similar to those observed in *vps29*, further demonstrating that the subunits of the core retromer are indispensable for the function of the retromer core complex in PIN endocytic cycling and plant development (Zelazny *et al*., 2013). Indeed, tandem affinity purification (TAP) with VPS29 finds that the other components include VPS35a, VPS35b, VPS35c, VPS26a and VPS26b (Nodzyński *et al*., 2013). VPS29 has been confirmed to natively co‐immunoprecipitate with VPS35a in the cytosol of plant cells, suggesting that the core retromer complex is first formed in the cytosol and is then recruited to the membrane of the endosomes (Jaillais *et al*., 2007). These biochemical data indicate that the composition of the subunits in the core retromer is conserved in eukaryotes. However, compared to mammals and yeasts that contain > 30 and about 10 SNX proteins, respectively, only three SNXs including SNX1, SNX2a and SNX2b have been characterized in *Arabidopsis* (Pourcher *et al*., 2010). SNX1 colocalizes with VPS29 to the same endosomes (Jaillais *et al*., 2007). In the *vps29* mutant, the SNX1‐GFP‐labeled endosomes display aberrant enlarged morphology (Jaillais *et al*., 2007). The *snx1* or *snx2a* mutants display weak developmental phenotypes, whereas both of them interact synergistically with *vps29,* and *snx1 vps29* or *snx2a vps29* double mutants are lethal, indicating that the SNXs work together with VPS29 in the same plant developmental pathways (Pourcher *et al*., 2010). These findings demonstrate that the normal morphology of the LE/MVB/PVC and the retromer function are pivotal for PIN endocytic trafficking and plant development. However, the regulation of them during plant development is still unclear.

In yeast, Vps38p forms a complex with Vps30p, Vps15p and Vps34p to regulate retrograde transport by generating a specific pool of phosphatidylinositol 3‐phosphate (PtdIns3P) for the function of the retromer complex (Burda *et al*., 2002). In animals, the PtdIns3P generated by the VPS34 complex is also central to the regulation of retromer function. For example, the murine VPS34 promotes the recycling of the IL‐7Rα in the T lymphocyte by affecting the proper location of VPS36 and the retromer function (McLeod *et al*., 2011). More recently, the interaction of UVRAG and BECLIN 1/VPS30 has been proposed to be essential for the roles of BECLIN 1/VPS30 during the control of PtdIns3P distribution, late endosome formation and endocytosis in murine neurons (McKnight *et al*., 2014). These data indicate that VPS38/UVRAG plays a pivotal role in the regulation of retromer function and late endosome formation by associating with the VPS34 complex in both yeasts and animals.

We conducted a genetic screen for mutants with defects in leaf development using the sets of confirmed SALK lines. We obtained a leaf defective mutant denominated *usl1* because we first observed that the mutant produced **u**nflattened and **s**mall **l**eaves. Further analysis showed that *usl1‐1* displayed pleiotropic developmental phenotypes. Genetic analysis confirmed that the disruption of *USL1* function causes the developmental defects in *usl1‐1*. *USL1* encodes a protein with a domain structure similar to those in the yeast Vps38p and human UVRAG. We demonstrate that auxin homeostasis and the subcellular distribution of the PIN1 proteins are disrupted in *usl1‐1*. USL1 colocalizes with VPS29 in the LE/MVB/PVC, and the VPS29‐associated LE/MVB/PVC becomes enlarged and circular in *usl1‐1*. We further demonstrate that USL1 interacts with AtVPS30 *in vivo* and *in vitro*. Our findings suggest that USL1 controls plant development by forming a complex with AtVPS30 and AtVPS34 to regulate late endosome morphology.

## Materials and Methods

### Plant materials and growth conditions

The model plant species *Arabidopsis thaliana* (L.) ecotype Columbia‐0 (Col‐0) was used. To carry out a screen for leaf defective mutants, we grew the sets of confirmed SALK lines (CS27941, CS27942 CS27943 and CS27944) one by one and observed the leaf defective phenotypes during plant growth. From the sets of CS27943, we found that *SALK_094540* produced **u**nflattened and **s**mall leaves, thus named it as *usl1‐1*. The *usl1‐2 (SAIL_552_F02)* was ordered from a public database. *Arabidopsis* seeds from the wild‐type (WT), mutants, transgenic plants and crossed plants were placed on 0.5× Murashige and Skoog (MS) medium with or without 50 μg ml^−1^ or kanamycin 20 μg ml^−1^ dl‐phosphinothricin. The plates were kept at 4°C for 3 d to stratify seeds before being placed at 22°C under long‐day conditions (16 h : 8 h, light : dark) for 7 d. Seedlings of *Arabidopsis* or *Nicotiana benthamiana* were transferred to soil and grown under the same conditions as described above.

### Genotyping analysis and gene expression assay

All of the primers used in this study are listed in Supporting Information Table S1.

The genotyping of *SALK_094540* (*usl1‐1*) was performed using *usl1‐1*‐LP, *usl1‐1*‐RP, LBb1.3 primers. *SAIL_552_F02* (*usl1‐2*) was genotyped using *usl1‐2*‐LP, *usl1‐2*‐RP and LB3 primers. The *pin1*‐LP, *pin1*‐RP and LBb1.3 primers were used to genotype *SALK_047613* (*pin1*).

For semi‐quantitative PCR, the total RNA from the WT and *usl1‐1* seedlings were extracted using a plant total RNA purification kit (GeneMark, Taichung, Taiwan, cat. no. TR02‐150). RNA was treated with a DNase as the protocol described in the RNA purification kit, and was then reverse transcribed using an M‐MLV kit (Promega, cat. no. A5003) in a reaction volume of 20 μl. The cDNA was diluted and used as a template for semi‐quantitative PCR. The cycling conditions of genotyping PCR were 94°C for 30 s, 55°C to 58°C for 30 s, and 72°C for 60 s to 120 s with 30 cycles, whereas semi‐quantitative PCR was limited to 28 cycles with the above conditions.

### Generation of binary constructs and transformation

The coding sequences of VPS29, AtVPS30, AtVPS34 and USL1 were amplified from *Arabidopsis* seedling cDNA. The products were cloned into pENTR/D‐TOPO (Invitrogen) to generate pENTRY‐VPS29, pENTRY‐VPS30, pENTRY‐VPS34 and pENTRY‐USL1.

In order to generate the complementary construct USL1pro‐USL1‐GFP, the genomic sequence was amplified from the *Arabidopsis* genomic DNA using the primers USL1proF/USL1Rnsc. The products were cloned into pENTR/D‐TOPO to generate pENTRY‐USL1pro‐USL1nsc. USL1pro‐USL1‐GFP was generated by an LR reaction between pENTRY‐USL1pro‐USL1nsc wand pK7FWG0.

In order to examine the USL1 expression pattern, a 969‐bp promoter region upstream of the ATG of USL1 was amplified from the *Arabidopsis* genomic DNA using primers USL1ProF/USL1ProR. The fragment was cloned into pENTR/D‐TOPO (Invitrogen) to generate pENTRY‐USL1pro. USL1pro‐GUS was generated by an LR reaction between pENTRY‐USL1pro and pKGWFS7.

In order to observe the subcellular localization of VPS29, the coding sequences of VPS29 without the stop codon were amplified using the primers VPS29F/VPS29Rnsc. The fragment was cloned into pENTR/D‐TOPO to generate pENTRY‐VPS29. 35S‐VPS29‐RFP was generated by an LR reaction between pENTRY‐VPS29 and pB7RWG2.

In order to observe the truncated USL1 subcellular localization, the truncated genomic sequence USL1pro‐USL1▵CC1 for complememntation was amplified from the *Arabidopsis* genomic DNA by overlap extension PCR using primers USL1proF/USL1▵CC1genomR and USL1▵CC1genomF/USL1Rnsc. The primers USL1proF/USL1▵CC1genomR and USL1▵CC1genomF/USL1Rnsc were used for the first round of PCR, and the primers USL1proF/USL1Rnsc were used for the second PCR using the first round PCR production as template. The same strategy was adopted to amplify USL1pro‐USL1▵CC2 using primers USL1proF/USL1▵CC2genomR, USL1▵CC2genomF/USL1Rnsc. The products of the second PCR were cloned into pENTR/D‐TOPO to generate pENTRY‐USL1pro‐USL1▵CC1 and pENTRY‐USL1pro‐USL1▵CC2. The USL1pro‐USL1▵CC1‐GFP and USL1pro‐USL1▵CC2‐GFP were generated by LR reactions between pK7FWG0 and pENTRY‐USL1pro‐USL1▵CC1 or pENTRY‐USL1pro‐USL1▵CC2.

In order to generate 35S‐AtVPS30‐FLAG and 35S‐USL1‐MYC, the full‐length coding regions of AtVPS30 and USL1 without a stop codon were amplified using primers VPS30F/VPS30Rnsc and USL1F/USL1Rnsc, respectively. The fragments were cloned into pENTR‐D/TOPO to generate pENTRY‐VPS30nsc and pENTRY‐USL1nsc. LR reactions were conducted between pENTRY‐VPS30nsc and pB7FLAGWG2 to generate 35S‐AtVPS30‐FLAG. pENTRY‐USL1nsc was cloned into pK7MYCWG2 by an LR reaction to generate 35S‐USL1‐MYC.

Constructs were transformed into *Agrobacterium tumefaciens* GV3101 and then into *Arabidopsis* as described previously (Qin *et al*., 2005).

### GUS staining and venation observation

The β‐Glucuronidase (GUS) staining assay was conducted as described previously (Zhang *et al*., 2017).

For venation observation, the leaves from 21‐d‐old plants were fixed with an ethanol : acetic acid (6 : 1) mixture for 24 h. The samples were treated with 100% ethanol for 30 min twice and once with 70% ethanol for 30 min. The samples were then immersed in a chloral hydrate : glycerol : H_2_O (8 g : 1 ml : 2 ml) mixture for at least 24 h. The venations were observed using a Leica M205FA stereoscope.

### Subcellular localization observation and chemical treatments

The complementary transgenic T_3_ plants with USL1pro‐USL1‐GFP in *usl1‐1* were crossed with the endomembrane marker lines (wave line markers) (Geldner *et al*., 2009). For the colocalization of VPS29 and USL1, 35S‐VPS29‐RFP was transformed into complementary transgenic T3 plants with USL1pro‐USL1‐GFP in *usl1‐1*.

The fluorescence was observed with a Zeiss LSM 710 NLO. To quantify the colocalization, Pearsons Correlation and scatterplot were generated using the Volocity software. At least 20 individual cells for each experiment were chosen for the calculations.

For the observation of different endomembrane markers in the *usl1*, the Wave line was crossed with both *usl1‐1* and *usl1‐2*. At least 10 independent lines were observed to confirm the results.

For the Wortmannin treatment, the 5‐d‐old crossed seedlings of USL1‐GFP with RABF2a‐mCherry were used. The seedlings were treated with 33 μM Wortmannin (Invitrogen, 3.3 mM stock in DMSO, diluted with deionized and distilled water) for 1 h, and the control was treated with the same concentration of DMSO diluted with deionized and distilled water.

### Scanning electron microscopy

The fifth leaves from 21‐d‐old WT and *usl1‐2* plants were isolated. Scanning electron microscopy (SEM) was conducted as described previously (Tao *et al*., 2013). Leaves were observed using a scanning electron microscope (Jeol JSM6610LV) following the manufacturer's instructions. The areas of leaf epidermal cells were analyzed using imagej software, and the frequency of cells sizes was calculated.

### Co‐IP with mass spectrometry assay

Approximately 3 g leaf tissues from 20‐d‐old 35S‐USL1‐MYC and 35S‐VPS30‐FLAG transgenic plants were ground in liquid N_2_. The proteins were extracted and purified using the method described previously by Li *et al*. (2015). The purified proteins were separated using SDS‐PAGE. The entire gel lane was excised and dehydrated. Proteins were digested in‐gel with nedoproteinase trypsin (0.5 ng μl^−1^ trypsin in 50 mM ammonium bicarbonate, pH 8.5). The extracted peptides were sequenced by LC‐MS/MS using a Velos Pro Orbitrap Elite mass spectrometer (Thermo Scientific, Waltham, MA, Waltham, MA, USA) equipped with a nano‐ESI source. The IPI (International Protein Index) *Arabidopsis* protein database was used as a searching platform.

### Firefly luciferase complementation imaging assay

In order to test the interaction of AtVPS30 and USL1 using the firefly luciferase complementation imaging assay, the pCAMBIA‐USL1‐nLUC was generated by an LR reaction between pCAMBIA‐nLUC (Chen *et al*., 2007; Zhang *et al*., 2017) and pENTRY‐USL1nsc. The pCAMBIA‐cLUC‐VPS30 was generated by an LR reaction between pCAMBIA‐cLUC (Zhang *et al*., 2017) and pENTRY‐VPS30nsc.

The constructs were transformed into *Agrobacterium tumefaciens* GV3101. The different combinations were co‐infiltrated into *N. benthamiana* leaves. The leaves were observed under a low‐light cooled CCD imaging apparatus (Lumazone 1300B; Roper Bioscience).

### Split ubiquitin Y2H assay

For the yeast two‐hybrid (Y2H) assay, the truncated USL1▵C and USL1▵N were amplified using the primers USL1F/USL1▵CR and USL1▵NF/USL1Rnsc. The USL1▵CC1, USL1▵CC2 were amplified by overlap extension PCR. The first PCR was conducted by primers USL1F/USL1▵CC1R, USL1▵CC1F/USL1Rnsc and USL1F/USL1▵CC2R, USL1▵CC2F/USL1Rnsc, respectively. The second PCR was then amplified using the primers USL1F/USL1Rnsc. The fragments were cloned into pENTR/D‐TOPO to generate pENTRY‐USL1▵N, pENTRY‐USL1▵C, pENTRY‐USL1▵CC1 and pENTRY‐USL1▵CC2. All of the pENTRY vectors were cloned into MetYCgate by the LR reaction to generate USL1‐Cub, USL1▵N‐Cub, USL1▵C‐Cub, USL1▵CC1‐Cub and USL1▵CC2‐Cub. pENTRY‐VPS30 and pENTRY‐VPS34 were cloned into pPR3‐N using the LR reaction to generate NubG‐VPS30 and NubG‐VPS34. pPR3‐NubWT was used as the positive control, and pPR3‐NubG was used as the negative control. The different combinations were cotransformed into the yeast strain NMY51 (Biotech), respectively.

Medium SD‐Trp‐Leu‐His‐Ade was used to vertify the interaction between the different combinations.

### RNA‐sequencing (RNA‐seq) analysis

Total RNAs were extracted from 7‐d‐old seedlings of *usl1‐2* or WT control using an RNA purification kit (GeneMark, Cat. no. TR02‐150). The RNA samples were used to perform RNA‐seq on Illumina Hi‐seq 2500 sequencer in the Biodynamic Optical Imaging Center (BIOPIC) of Peking University. tophat v.2.0.14 (Kim *et al*., 2013) was used to map reads with the Arabidopsis genome in TAIR10 (http://www.arabidopsis.org/download_files/Genes/TAIR10genomerelease/TAIR10chromosomefiles/TAIR10_chr_all.fas). The cuffdiff (v.2.2.1; Trapnell *et al*., 2012) was used for stringent statistical analysis to normalize and find the differential expression levels of the RNAs using the FPKM values and the *P*‐values in the output files. Gene ontology enrichment analysis was conducted on BMKcloud website (http://www.biocloud.net/). Part of the analysis was performed on the Computing Platform of the Center for Life Science of Peking Univerisity.

## Results

### Identification of the *usl1* mutants

**Figure 1:**
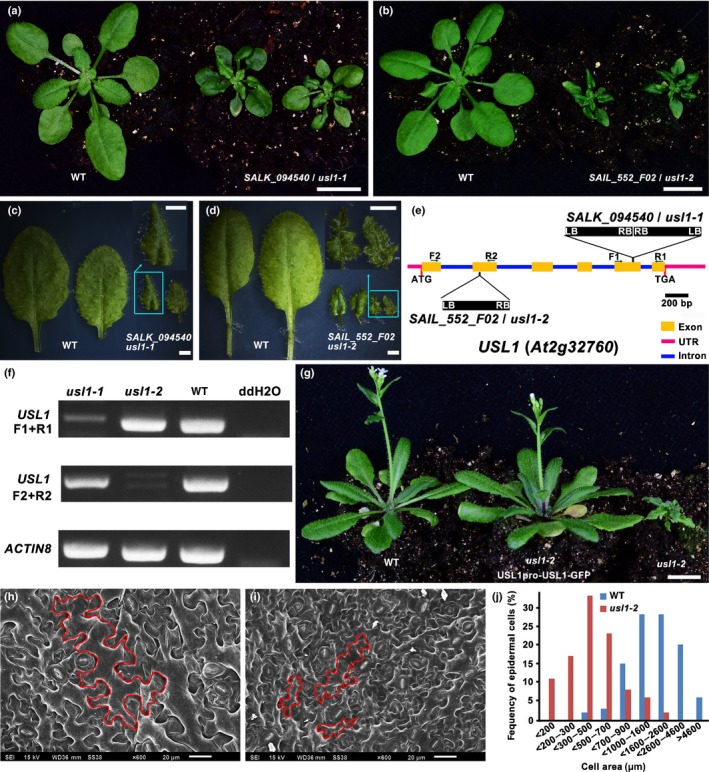
The mutant ***u**nflattened and **s**mall **l**eaves* (*usl1‐1* and *usl1‐2*) display severe leaf phenotypes in Arabidopsis. (a) The phenotypes of 21‐d‐old wild‐type (WT) control and *usl1‐1*. (b) The phenotypes of 21‐d‐old WT control and *usl1‐2*. (c, d) Close‐up views of the 7^th^ and the 8^th^ leaves from the 21‐d‐old WT control and the *usl1‐1* mutant. (e) Schematic representation of the T‐DNA insertion sites in the *usl1‐1* and *usl1‐2* mutants. The F1 and R1 or F2 and R2 primer pairs used for the gene expression and genotyping analysis of *usl1‐1* or *usl1‐2*, respectively, are indicated by the black lines with arrows. LB, T‐DNA left border; RB, T‐DNA right border. (f) Semi‐quantitative polymerase chain reaction analysis of *usl1‐1* and *usl1‐2* showed that *usl1‐1* and *usl1‐2* were null mutants. *ACTIN8* gene was used as a control. (g) Complementation of *uls1‐2* leaf phenotypes by USL1pro‐USL1‐GFP. (h, i) Scanning electron micrographs of the leaf epidermal cells of the 5^th^ leaf from (h) 21‐d‐old WT, and (i) 21‐d‐old *usl1‐2*. (j) Distribution of cell size of the leaf epidermal cells from *usl1‐2* and the WT control. Bars: (a, b, g) 1 cm; (c, d) 1 mm; (h, i) 20 μm.

In order to find regulators that control leaf development, we screened the sets of confirmed SALK lines for leaf defective mutants. In this study, we report a mutant denominated *usl1‐1* because it produces unflattened and small leaves (Fig. 1a,c). Compared to that of the WT control, *usl1‐1* produces leaves with wavy margins and uneven surfaces (Fig. 1a,c). The SALK number for *usl1‐1* is *SALK_094540* that contains two T‐DNA sequences inserted head to head in the fifth exon of *At2g32760* (Fig. 1e). The T‐DNA insertions disrupted the expression of *At2g32760* in *usl1‐1* when we used the primer pair (F1 and R1) designed from the flanking sequences of T‐DNA insertion site (Fig. 1e,f), because no specific bands were amplified. However, the truncated transcripts of *At2g32760* were found in *usl1‐1* using the primer pair (F2 and R2) designed from the sequence upstream of T‐DNA insertion site in *usl1‐1* (Fig. 1f). To test whether the disruption of *At2g32760* function could lead to the small and curled leaves in *usl1‐1*, we first identified the mutant allele *SAIL_552_F02* in which the T‐DNA was inserted in the second exon of *At2g32760* (Fig. 1e). No specific bands were found when testing the expression of *At2g32760* using F2 and R2 primers designed from the the flanking sequences of T‐DNA insertion site in *SAIL_552_F02* (Fig. 1e,f). However, the truncated transcripts of *At2g32760* were also observed when using F1 and R1 primers (Fig. 1e,f). The mutant displayed the small and curled leaf phenotypes resembling those observed in *usl1‐1* (Fig. 1b,d,f), We thus designated *SAIL_552_F02* to be *usl1‐2*. However, the phenotypes of *usl1‐2* were stronger than that of *usl1‐1*, suggesting that the truncated transcripts of *At2g32760* in *usl1‐1* might be partially functional (Fig. 1a–e). We then generated the construct USL1pro‐USL1‐GFP in which *At2g32760* was fused to the gene encoding the green fluorescent protein (GFP) and was driven by its own promoter. We transformed the construct into the heterozygous *usl1‐1* or *usl1‐2* because the homozygous *usl1* mutants also displayed dwarfism and low fertility as described below. The analysis of transgenic lines indicated that the expression of USL1‐GFP completely complemented the developmental defects of the *usl1* mutants (Figs 1g, S1). We thus designated At2g32760 to be USL1. In addition, we found that the leaf epidermal cells in *usl1‐2* were obviously smaller than those in the WT control (Fig. 1h–j). These data demonstrate that *USL1* significantly affects leaf development by affecting cell expansion.

*USL1* encodes a predicted protein containing 352 amino acid residues. Bioinformatical analysis suggests that USL1 shares 23.9% similarity with the yeast Vps38p and 20.3% similarity with the human UVRAG in its protein sequence. We further used the Protein Homology/analogY Recognition Engine v.2.0 (PHYRE) tool to predict the USL1 protein structure (Kelley *et al*., 2015). The results suggested that USL1 was homologous to the yeast Vps38p (Fig. S2a). The protein modeling of the USL1 structure based on the yeast Vps38p template suggested that USL1 contains two predicted CC (coiled coil) domains in the N‐terminus and a BARA (β‐α repeated, autophagy‐specific) domain in the C‐terminus (Fig. S2b,c). These data suggest that USL1 could be structurally orthologous to Vps38p or UVRAG.

### The *usl1* mutants display pleiotropic developmental phenotypes

**Figure 2:**
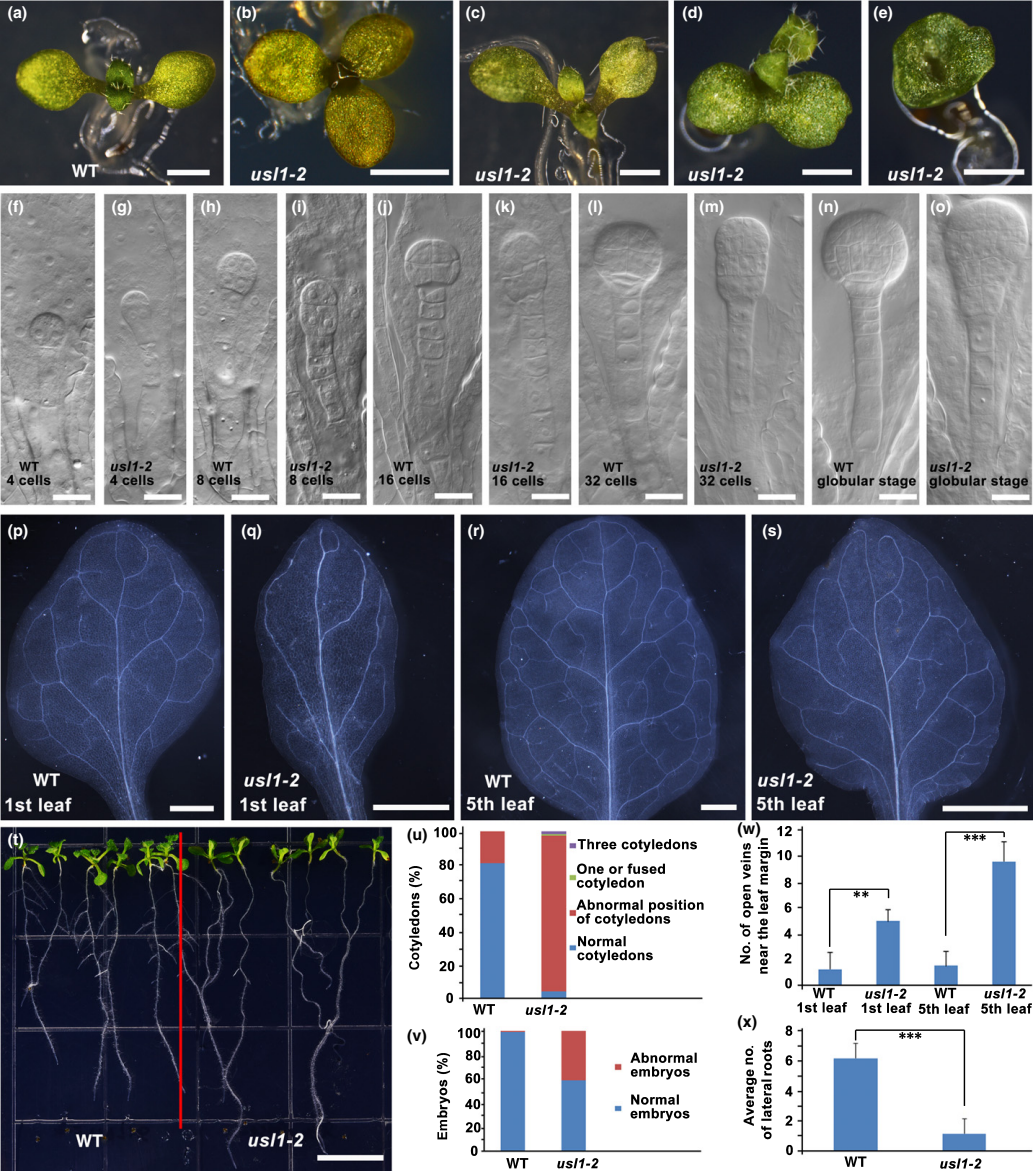
The ***u**nflattened and **s**mall **l**eaves* (*usl1*) mutants multiple developmental phenotypes in addition to leaf defects in Arabidopsis. (a–e) The cotyledons of (a) 10‐d‐old wild‐type (WT) seedling and (b) *usl1* seedlings with three cotyledons, (c, d) with abnormal position of cotyledons, or (e) with fused cup‐shaped cotyledons. (f–o) Early embryogenesis from the four‐cell stage to the globular stage in (f, h, j, l, n) the WT and (g, i, k, m, o) *usl1‐2* mutant. The developmental stages are indicated at the bottom of each picture. (p–s) The vasculature of the 1^st^ and 5^th^ leaf from 21‐d‐old (p, r) WT, or (q, s) *usl1‐2* plants. (t) The 15‐d‐old *usl1‐2* mutant produced fewer secondary roots than the WT. (u) The statistical analysis of abnormal cotyledons in (a–e) (*n *=* *116). (v) The statistical analysis of abnormal embryos in (f–o) (WT, *n *=* *147; *usl1‐2*,* n *=* *162). (w) The statistical analysis of open veins near the leaf margins in (p–s). Data are means ± SD (*n *=* *8), (Student's *t*‐test): **, *P *<* *0.005; ***, *P *<* *0.001. (x) The statistical analysis of lateral roots in (t). Data are means ± SD (*n *=* *12), (Student's *t*‐test): ***, *P *<* *0.001. Bars: (a–e, p–s) 1 mm; (f–o) 20 μm; (t) 1 cm.

In addition to leaf developmental defects, the *usl1* mutant also displayed other pleiotropic developmental phenotypes. First, the *usl1‐2* seedlings produced cotyledons abnormal in their number, position and shape (Fig. 2a–e,u). Second, in consistent with the abnormal cotyledons, the cell division and embryogenesis were compromised in the *usl1‐2* mutant (Fig. 2f–o,v). Third, the leaf venation of *usl1‐2* exhibited a simpler pattern with more open veins near the leaf margins displayed fewer secondary or higher order veins than those of the wild‐type control (Fig. 2p–s,w). Fourth, the *usl1‐2* mutant produced fewer lateral roots than wild‐type control (Fig. 2t,x). Finally, the *usl1‐2* mutant also displayed late flower, low fertility and abnormal silique phyllotaxy (Fig. S3). These data indicate that USL1 regulates multiple aspects during plant development.

### *USL1* is expressed in multiple organs

**Figure 3:**
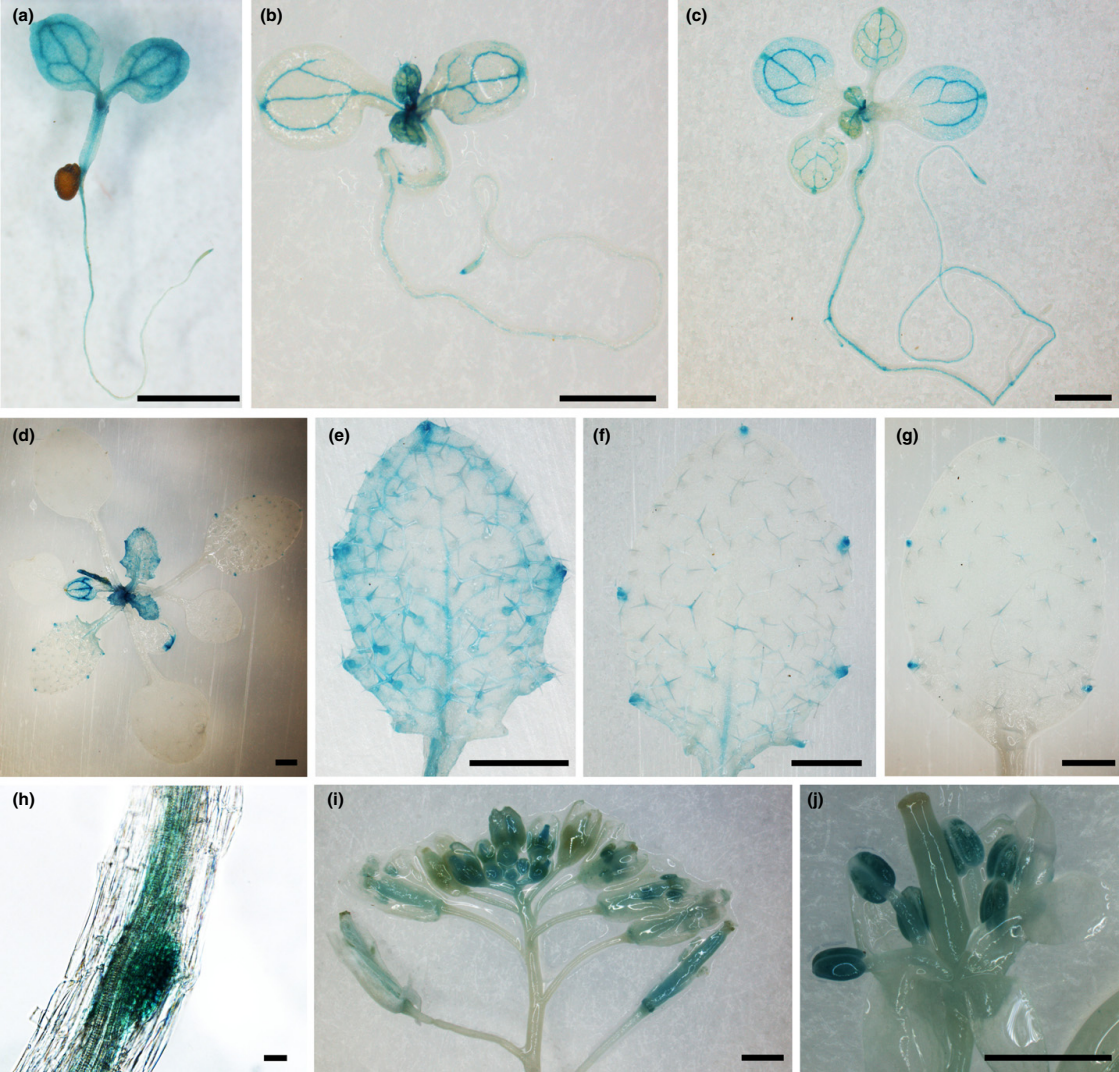
The Arabidopsis ***U**NFLATTENED AND **S**MALL **L**EAVES* (*USL1*) is expressed in multiple organs. (a–g) β‐Glucuronidase (GUS) staining of the USL1pro‐GUS transgenic plants. (a) Seven‐day‐old seedling. (b) Ten‐day‐old seedling. (c) Fourteen‐day‐old plant. (d) Nineteen‐day‐old plant. (e–g) Close up views of (e) the 4^th^ leaf, (f) the 3^rd^ leaf, and (g) the 2^nd^ leaf from a 19‐d‐old plant (d). (h) Close‐up view of lateral root initial site. (i) GUS staining of the inflorescence. (j) Close‐up view of a dissected flower. Bars: (a–g, i, j) 1 mm; (h) 20 μm.

In order to identify the expression pattern of *USL1*, we cloned the 969 bp *USL1* promoter from the TAA of the *USL1* upstream gene to the start codon of *USL1*. We generated an USL1pro‐GUS construct in which the β‐glucuronidase (*GUS*) gene was driven by the *USL1* promoter. Sixteen USL1pro‐GUS transgenic plants showed similar GUS staining patterns. Histochemical analysis showed that *USL1* was predominantly expressed in the shoot apical meristem (SAM) and in the leaf vascular tissues (Fig. 3a–d). Strong GUS activity was observed in the nascent leaves. Interestingly, as the leaves grew, the GUS staining was focused in the leaf vasculature and became stronger at the leaf tips or the hydathodes along the leaf margins (Fig. 3d–g). Strong GUS staining was also observed in the root tip, root vasculature, lateral root initiation sites, anther, pollen and pistils (Fig. 3a–c,h–j). These data demonstrate that *USL1* is expressed in different organs. This is consistent with the observed pleiotropic developmental phenotypes in *usl1*, and the expression pattern suggests that *USL1* plays an important function in plant development.

### USL1 is localized to the LE/MVB/PVC

**Figure 4:**
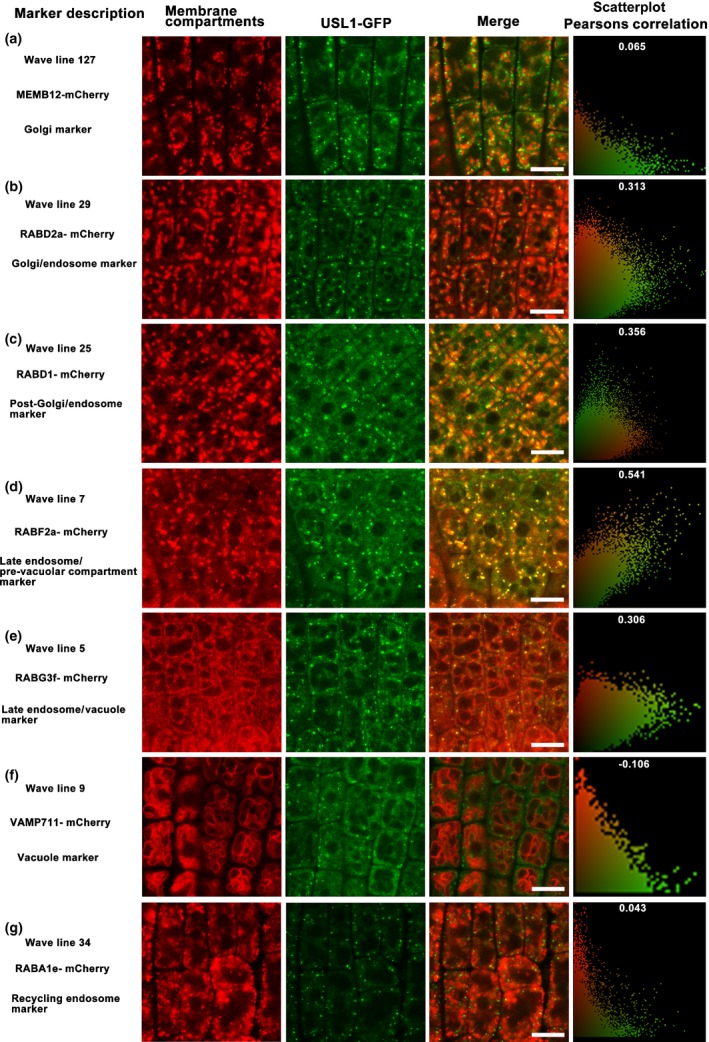
The Arabidopsis **U**nflattened and **S**mall **L**eaves (USL1) is localized to the late endosomes/multivesicular bodies/prevacuolar compartments (LE/PVC/MVB). The mCherry‐Wave (R) marker lines were crossed with *usl1‐1* mutant complemented by USL1pro‐USL1‐GFP. (a) The fluorescence of the Golgi marker MEMB12‐mCherry and USL1‐GFP. (b) The fluorescence of the Golgi/endosome marker RABD2a‐mCherry and USL1‐GFP. (c) The fluorescence of the post‐Golgi/endosome marker RABD1‐mCherry and USL1‐GFP. (d) The fluorescence of the late endosome/multiple vesicle body/prevacuolar compartments (LE/MVB/PVC) marker RABF2a‐mCherry and USL1‐GFP. (e) The fluorescence of the LE/vacuole marker RABG3f‐mCherry and USL1‐GFP. (f) The fluorescence of the vacuole marker VAMP711‐mCherry and USL1‐GFP. (g) The fluorescence of the recycling endosome marker RABA1e‐mCherry and USL1‐GFP. Scatterplots of the fluorescence values of the pixels of the two channels are provided. To quantify the colocalization, the Pearsons Correlation was calculated after analyzing the cytosolic areas of at least 20 cells. GFP, green fluorescent protein. Bars, 10 μm.

In order to determine the subcellular localization of USL1, we crossed *usl1‐1* complemented by USL1pro‐USL1‐GFP to a series of mCherry‐Wave (R) marker lines indicating different plant inner membrane compartments from the Golgi to the vacuole (Geldner *et al*., 2009). Our observations showed that the USL1‐GFP‐labeled compartments did not co‐localize with the Golgi indicated by MEMB12‐mCherry or the Golgi/endosome by RabD2a‐mCherry (Fig. 4a,b). However, USL1 proteins could be seen close to the Golgi or Golgi/endosome. Further analysis showed that the USL1‐associated compartments highly overlapped the RABF2a‐mCherry‐labeled LE/MVB/PVC (Fig. 4d; Movies S1), whereas it only partially overlapped the RabD1‐mCherry‐labeled post‐Golgi/endosome (Fig. 4c) and RabG3f‐mCherry‐labeled late endosome/vacuole (Fig. 4e), respectively. USL1 was not localized to the vacuoles labeled by VAMP711‐mCherry and the recycling endosomes labeled by RabA1e‐mCherry (Fig. 4f,g). These data suggest that USL1 exerts its main function at the sites of the late endosomes/multivesicular bodies/prevacuolar compartments (LE/MVB/PVC) membranes.

### USL1 is essential to maintain the morphology of the VPS29‐associated endosomes

**Figure 5:**
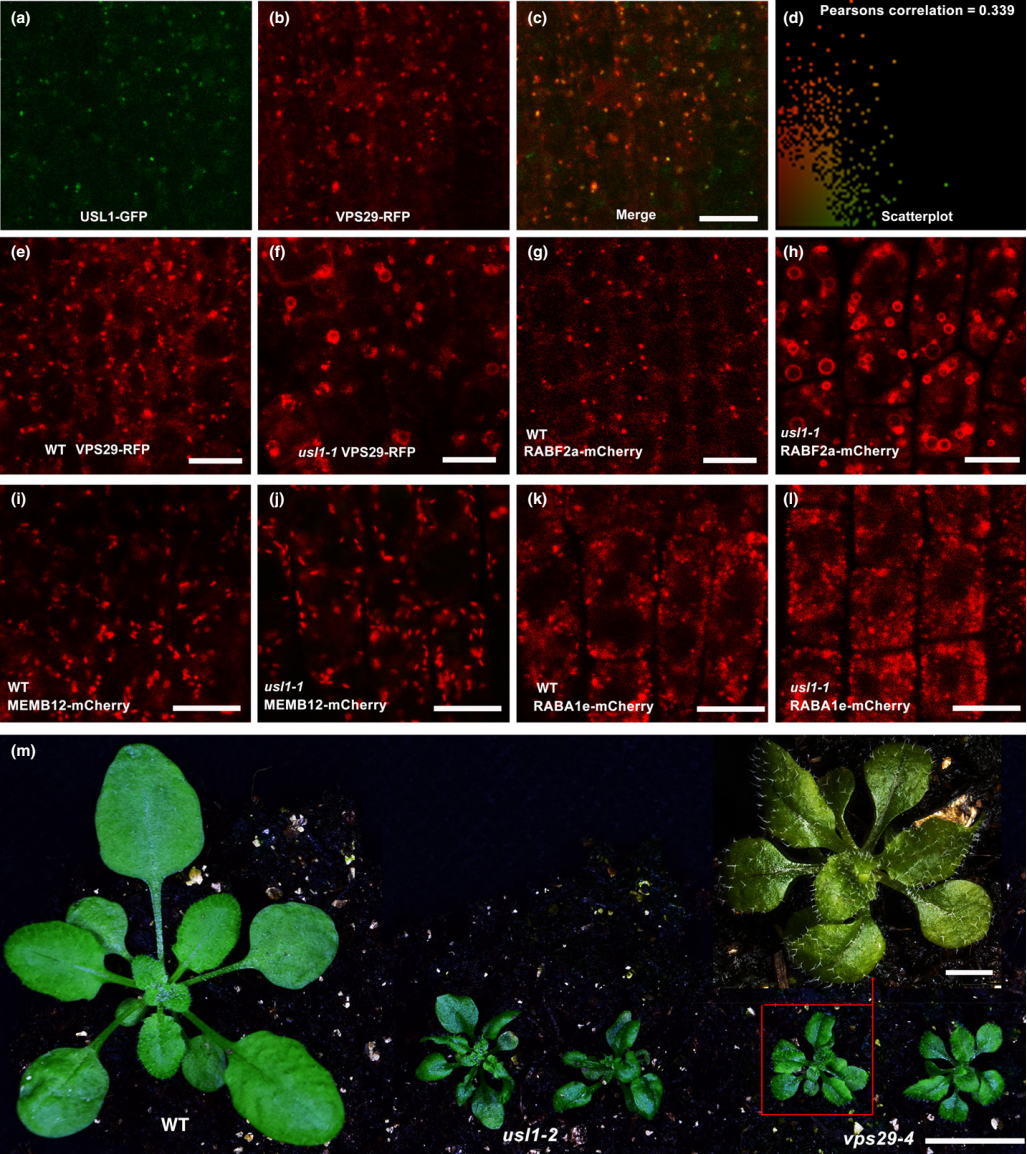
The Arabidopsis **U**NFLATTENED AND **S**MALL **L**EAVES (USL1) controls the morphology of VPS29‐related late endosomes (VPS, VACUOLAR PROTEIN SORTING). (a–d) USL1‐GFP was colocalized with VPS29‐RFP. (e, f) The morphology of VPS29‐RFP‐labeled endosomes in (e) wild‐type (WT) plants and (f) *usl1‐1* mutants. (g, h) The morphology of RABF2a‐mCherry‐labeled endosomes in (g) WT plants and (h) *usl1‐1* mutants. (i, j) the MEMB12‐mCherry‐labeled endomembrane compartment in (i) WT plants and (j) *usl1‐1* mutants. (k, l) RABA1e‐mCherry‐labeled endomembrane compartment in (k) WT plants and (l) *usl1‐1* mutants. (m) Both *usl1‐2* and *vps29‐4* produced small and curled leaves. RFP, red fluorescent protein; GFP, green fluorescent protein. Bars: (a–l) 10 μm; (m) 1 cm; (m inset) 1 mm.

The retromer component VPS29 has been shown to strongly colocalize with the mRFP‐RABF2b‐labeled endosome (Jaillais *et al*., 2007), whereas our data showed that USL1 localized to the RABF2a‐mCherry labeled endosome. RABF2a and RABF2b are canonical orthologs of animal RAB5 in *Arabidopsis*, and both are localized to the LE/MVB/PVC (Ueda *et al*., 2004). This implies that USL1 could be colocalized with VPS29 in the LE/MVB/PVC. To test this hypothesis, we expressed VPS29‐RFP in *usl1‐1* complemented by USL1pro‐USL1‐GFP. The results clearly showed that the USL1‐GFP‐labeled endomembrane compartments largely overlapped with the VPS29‐RFP‐associated endosomes (Fig. 5a–d; Movies S2), suggesting that USL1 could regulate the function of the retromer. We then investigated the possible changes of the VPS29‐RFP‐labeled compartments in *usl1‐1*. Interestingly, compared to the small dot morphology of VPS29‐RFP‐labeled endosomes in the WT control, the loss of *USL1* function in *usl1‐1* caused the VPS29‐RFP‐labeled endosomes to become enlarged circles (Fig. 5e,f). Similarly, RABF2a‐mCherry‐labeled endosomes also displayed enlarged round morphology in *usl1‐1*, whereas they were distributed in small dots in the WT control (Fig. 5g,h). However, the morphology of the Golgi labeled by MEMB12‐mCherry or the recycling endosomes by RABA1g‐mCherry in *usl1‐1* was not significantly different from those in the WT control (Fig. 5i–l). These results demonstrate that USL1 is specifically required to maintain the normal morphology of the VPS29‐associated LE/MVB/PVC.

The retromer mutant *vps29* displays severe auxin‐related phenotypes including abnormal cotyledons, defects in embryogenesis and fewer lateral roots as observed in *usl1‐2* (Jaillais *et al*., 2007; Fig. 2). The mutant allele *GK‐125H09* was previously reported to be a *vps29‐4* null mutant (Jaillais *et al*., 2007). To further show that *USL1* is related to *VPS29*, we first observed the leaves of *vps29‐4*. The *vps29‐4* mutant also produced small and uneven leaves similar to those observed in the *usl1* mutants (Fig. 5m). We then crossed *usl1‐2* to *vps29‐4*. We genotyped 170 progenies from the F_2_ population and found no *usl1‐2 vps29‐4* double mutant lines, suggesting that *usl1‐2 vps29‐4* is gametophytic or embryo lethal. The similar auxin‐related defects observed in both *usl1‐2* and *vps29‐4*, plus the synergistic genetic interaction between *usl1‐2* and *vps29‐4* further suggest that USL1 and VPS29 act in parallel in auxin polar transport.

### USL1 is required for PIN1 endocytic trafficking

**Figure 6:**
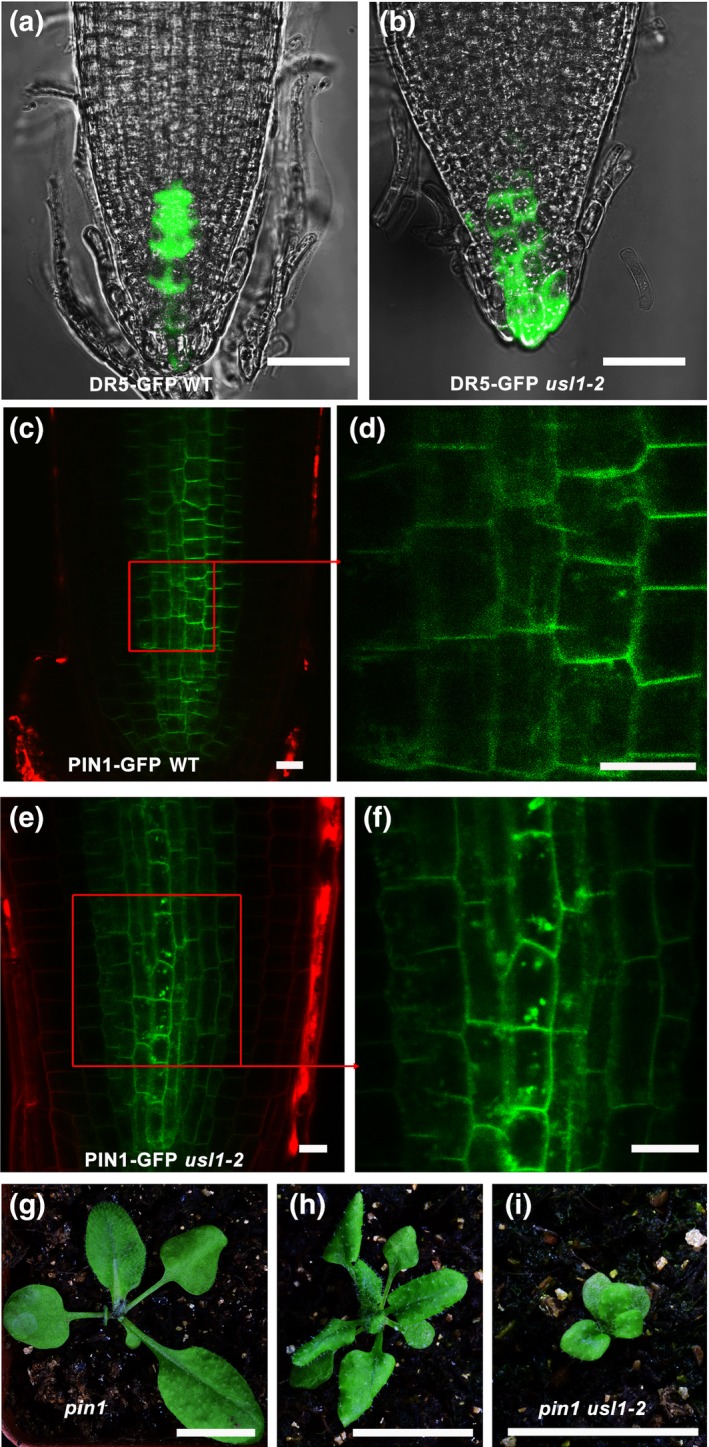
The Arabidopsis **U**NFLATTENED AND **S**MALL **L**EAVES (USL1) is required for PIN1 endocytosis. (a, b) The fluorescence of DR5:GFP in (a) wild‐type (WT) and (b) *usl1* primary roots. (c–f) Subcellular localization of PIN1‐GFP in (c, d) WT and (e, f) *usl1‐2*. (g–i) Twenty‐two‐day‐old (g) *pin1*, (h) *usl1‐2* and (i) *usl1‐2 pin1* double mutant. Bars: (a, b) 1 mm; (c–f) 10 μm; (g–i) 1 cm.

In order to provide more evidence for the hypothesis that USL1 could function in controlling auxin polar transport, we first crossed the auxin‐responsive DR5‐GFP reporter line with *usl1‐2* to examine the auxin distribution in *usl1‐2*. The results showed that the GFP signals were significantly reduced in the quiescent center (QC) but accumulated in the root cap in *usl1‐2* when compared to those in the WT control (Fig. 6a,b), indicating that auxin homeostasis was compromised in *usl1‐2*. We then crossed the PIN1‐GFP marker line to *usl1‐2*. Our observation clearly showed that the PIN1‐GFP signals were retained in the aberrant enlarged circular membrane compartments in *usl1‐2* (Fig. 6c–f), suggesting that USL1 was crucial for the proper PIN1 endocytic trafficking. In addition, we crossed *usl1‐2* to *SALK_047613*/*pin1*. The leaves of *usl1‐2 pin1* were even smaller than those of the single *usl1‐1* or *pin1* mutants (Fig. 6g–i), indicating that *usl1* synergistically interacted with *pin1*. These data continue to demonstrate that USL1 regulates the endocytic trafficking of PIN1 auxin transporter.

### USL1 interacts with the PI3K complex to regulate retromer function

In order to decipher the possible molecular mechanism by which USL1 regulates the morphology of the VPS29‐associated LE/PVC/MVB, we first generated the construct 35S‐USL1‐MYC in which the *USL1* fused with sequence encoding six MYC tags was driven by the CaMV 35S promoter. We used 35S‐USL1‐MYC transgenic plants as material to identify components of the USL1 complex by co‐immunoprecipitation coupled to mass spectrometry (Co‐IP/MS). The results showed that all of the main components of the PI3K complex including the AtVPS15, AtVPS30 and AtVPS34 isoforms were associated with USL1 (Table 1). To confirm that USL1 could interact with the PI3K complex, we generated 35S‐VPS30‐FLAG in which *AtVPS30* fused with sequence encoding three FLAG tags was driven by the CaMV 35S promoter. We performed Co‐IP/MS using 35S‐VPS30‐FLAG transgenic plants. The data showed that USL1, AtVPS15 and AtVPS34 all co‐purified with AtVPS30 (Table 2), indicating that USL1 did indeed form a complex with the PI3K components.

**Table 1:** Mass spectrometry analysis of proteins co‐immunoprecipitated with **U**NFLATTENED AND **S**MALL **L**EAVES (USL1)‐MYC in Arabidopsis

Protein accession	Gene ID	Gene product	Score	Mass	Spectra	Unique peptides	Seq. Cov (%)
IPI00516293	*At2g32760*	USL1	1462	40 062	49	19	62.8
IPI00532518	*At4g29380*	VPS15	752	170 070	27	19	18.7
IPI00521940	*At3g61710*	VPS30	546	59 151	22	11	26.5
IPI00517736	*At1g60490*	VPS34	64	94 294	2	2	2.5

**Table 2:** Mass spectrometry analysis of proteins co‐immunoprecipitated with VACUOLAR PROTEIN SORTING (VPS)30‐FLAG in Arabidopsis

Protein accession	Gene ID	Gene product	Score	Mass	Spectra	Unique peptides	Seq. Cov (%)
IPI00532518	*At4g29380*	VPS15	1416	170 070	53	45	41.5
IPI00517736	*At1g60490*	VPS34	820	94 294	24	20	34.4
IPI00521940	*At3g61710*	VPS30	802	59 151	25	21	41.4
IPI00516293	*At2g32760*	USL1	274	40 062	11	10	38.9

**Figure 7:**
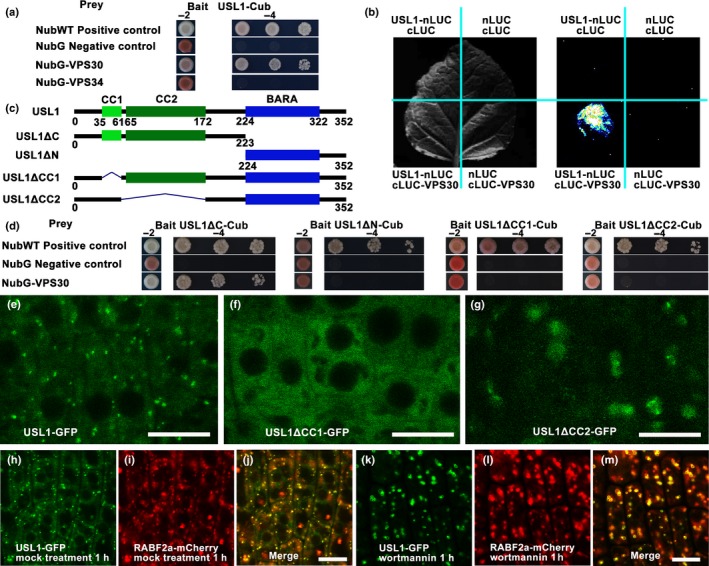
The Arabidopsis **U**NFLATTENED AND **S**MALL **L**EAVES (USL1) regulates the function of PI3K by forming a complex with AtVPS30 and AtVPS34 (VPS, VACUOLAR PROTEIN SORTING). (a) Yeast two‐hybrid (Y2H) assays of USL1 with AtVPS30 and AtVPS34. NubWT represents the wild‐type (WT) N‐terminal half of ubiquitin. NubG represents the mutated N‐terminal half of ubiquitin. Transformed yeasts were spotted on control medium (‐2: SD‐Leu‐Trp) or selective medium (‐4: SD‐Leu‐Trp‐His‐Ade) at dilutions of 10‐, 100‐, and 1000‐fold. (b) The firefly luciferase (LUC) complementation imaging assays show that USL1 interacted with AtVPS30. LUC signals were detected in the combination of USL1‐nLUC and cLUC‐AtVPS30, but not in the control combinations including USL1‐nLUC and cLUC, nLUC and cLUC‐AtVPS30 and nLUC and cLUC. (c) The schematic representation of the USL1 deletions. (d) Y2H assays between differently truncated USL1 and AtVPS30. (e–g) The subcellular location of (e) the USL1‐GFP protein, (f) USL1ΔCC1‐GFP protein, and (g) USL1ΔCC2‐GFP protein. (h–m) The morphological changes of late endosome/multiple vesicle body/prevacuolar compartments (LE/MVB/PVC) following treatment with the PI3K inhibitor Wortmannin. The fluorescence of (h) USL1‐GFP, (i) RABF2a‐mCherry and (j) the merged picture after the mock treatment. (k) The fluorescence of USL1‐GFP, (l) RABF2a‐mCherry and (m) the merged picture after treatment with Wortmannin. GFP, green fluorescent protein. Bars, 10 μm.

In order to determine which components directly interact with USL1, we performed split‐ubiquitin Y2H assays and found direct interactions between AtVPS30 and USL1 (Fig. 7a). The firefly luciferase complementation imaging assays again showed that USL1 was associated with AtVPS30 (Fig. 7b). To further identify the possible domains in USL1 responsible for the interaction with AtVPS30, we first generated two truncated fragments including USL1ΔC deleted C‐terminus and USL1ΔN deleted N‐terminus (Fig. 7c). The split‐ubiquitin yeast two‐hybrid assays showed that the N‐terminus was required for the interaction of USL1 and AtVPS30, whereas the BARA domain was not necessary for this interaction (Fig. 7d). The N‐terminus contained CC1 and CC2 domains (Fig. S2). We then generated the proteins with a truncated CC1 (USL1ΔCC1) or CC2 domain (USL1ΔCC2) (Fig. 7c). The results showed that the deletion of either CC1 or CC2 abolished the interaction between USL1 and AtVPS30 (Fig. 7d), indicating that both the CC1 and CC2 domains were essential for the association of USL1 with the PI3K complex.

In order to determine the biological function of the interaction between USL1 and the PI3K complex, we generated USL1pro‐USL1ΔCC1‐GFP and USL1pro‐USL1ΔCC2‐GFP constructs. The transgenic analysis showed that compared to the 87 lines among the *usl1‐1* mutants transformed with USL1pro‐USL1‐GFP that were recovered to normal phenotypes, none of the 117 *usl1‐1* mutants examined that were transformed with USL1pro‐USL1ΔCC1‐GFP or the 126 mutants with USL1pro‐USL1ΔCC2‐GFP were rescued, indicating that the association of USL1 with the PI3K complex was required for the biological function of USL1. We then investigated the subcellular localization of USL1ΔCC1‐GFP or USL1ΔCC2‐GFP. Interestingly, USL1ΔCC1‐GFP was no longer localized to the LE/PVC/MVB but was distributed evenly in the cytosol (Fig. 7e,f), whereas USL1ΔCC2‐GFP was found in an unknown compartment that was much larger than the LE/PVC/MVB (Fig. 7e,g). These results suggested that the USL1 interaction with the PI3K complex could be very important for USL1 to be localized to the LE/PVC/MVB. We then observed the localization of USL1‐GFP during treatment with Wortmannin, an inhibitor of PI3K activity. We found that the inhibition of PI3K led to a morphological change of the USL1‐associated LE/PVC/MVB that overlapped with the RABF2a‐mCherry‐labeled compartments (Fig. 7h–m). This is similar to the morphological change of RABF2a/VPS29‐associated LE/PVC/MVB in the *usl1* mutants (Fig. 5f,h). These results suggest that USL1 affects the PI3K function to regulate the retromer by direct association with AtVPS30.

### Genome‐wide transcriptome analysis of genes regulated by USL1

In order to further elucidate the mechanisms by which *USL1* regulates plant development, we performed a genome‐wide transcriptome analysis by RNA‐seq using the RNA samples from *usl1‐2* and WT control. The results showed that the expression level of 1826 genes was altered in *usl1‐2* when compared to WT control (fold change ≥ 2; *P*‐value < 0.05) (Notes S1, S2). Among them, 990 genes were upregulated and 836 ones were downregulated in *usl1‐2* mutant (Notes S1, S2). Gene ontology (GO) enrichment analysis showed that 114 biological processes were significantly upregulated and 129 ones were downregulated in *usl1‐2* (*P*‐value < 0.05) (Notes S3, S4). These altered biological processes include ‘response to auxin’, ‘leaf senescence’, ‘lateral root development’, ‘vacuolar protein processing’, ‘cytoplasm‐to‐vacuole targeting (Cvt) pathway’, ‘protein targeting to membrane’ and so on (Notes S3, S4). This is consistent with the multiple developmental phenotypes displayed in *usl1‐2*. We then searched our RNA‐seq data for auxin‐related genes. The results showed that the expression level of auxin‐related genes including many *SAUR* genes was altered in *usl1‐2* (Tables S2, S3). We further searched our RNA‐seq data for genes regulating leaf flattening or polarity. No leaf polarity‐related genes were significantly regulated in *usl1‐2* (Notes S1, S2). However, we found some leaf flattening‐related genes including *WUSCHEL‐RELATED HOMEOBOX1* (*WOX1*), *PRESSED FLOWER* (*PRS*)/*WOX3* and *TEOSINTE BRANCHED1*/*CYCLOIDEA*/*PCF* 17 (*TCP*) *TCP17* were downregulated in *usl1‐2* (Table S4) (Tao *et al*., 2013; Guan *et al*., 2017). Interestingly, we found *TCP Interactor containing EAR motif protein 1* (*TIE1*) and *TIE4* were upregulated in *usl1‐2*, consistent with the unflattened leaves in *usl1‐2* and the previous reports that the overexpression of *TIE1* or *TIE4* leads to leaves with wavy margins (Table S4) (Tao *et al*., 2013).

## Discussion

**Figure 8:**
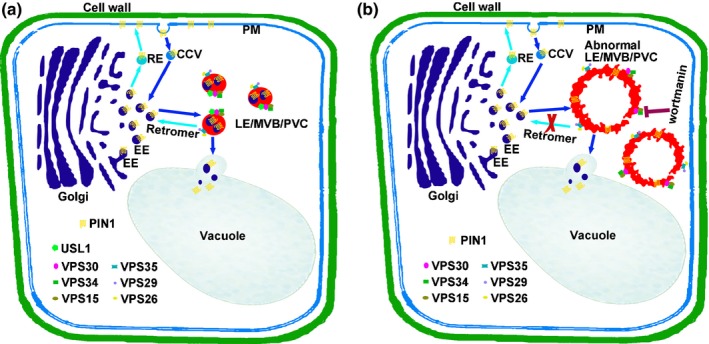
A work model of the Arabidopsis **U**NFLATTENED AND **S**MALL **L**EAVES (USL1). (a) In wild‐type cells, PIN1 proteins on plasma membrane (PM) can be internalized by membrane invagination to form clathrin‐coated vesicles (CCV). CCVs first reach early endosome (EE). Then from there, PIN1 proteins are sorted back to PM for reuse via recycling endosome (RE), or are delivered to vacuoles for degradation through late endosome/multiple vesicle body/prevacuolar compartments (LE/MVB/PVC). The retromer complex including VPS29, VPS26 and VPS35 acts at the LE/MVB/PVC to help PIN1 back to the EE for reuse. USL1 forms a complex with AtVPS15, AtVPS30 and AtVPS34 at the LE/MVB/PVC and regulates the morphology of LE/MVB/PVC. (b) In *usl1* mutant cells, the LE/MVB/PVC are abnormally enlarged and the retromer cannot function normally, so that PIN1 cannot recycle back to the EE.

In this study, we identified an important factor **U**NFLATTENED AND **S**MALL **L**EAVES (USL1) that plays essential roles in plant development by regulating retromer function. The expression pattern of *USL1* in different organs indicates that USL1 is important for plant organ development. The loss of *USL1* function in *usl1* mutants leads to small and uneven leaves by repressing leaf cell expansion. Our results also proved that *usl1* results in defects in leaf vein development. The subcellular localization of USL1 demonstrates that this factor acts at the RABF2a‐labeled endosome. USL1 interacts with the PI3K complex. The CC1 and CC2 domains at the N‐terminus of USL1 are essential for its subcellular localization and interaction with AtVPS30 in the PI3K complex. The disruption of USL1 in the *usl1* mutants causes enlarged aberrant VPS29‐associated endosomes. Based on these data, we propose a model for USL1 in regulating leaf development (Fig. 8). USL1 is recruited to the late endosomes/multivesicular bodies/prevacuolar compartments (LE/MVB/PVC) by interacting with AtVPS30, a key component of the PI3K complex. USL1 on the LE/MVB/PVC regulates the AtVPS34/PI3K function and possibly generates specific pools of PtdIns3P in the LE/MVB/PVC membrane to maintain the LE morphology and to cause the retromer to recycle the PIN1 proteins to the TGN. In the *usl1* mutants, the lack of USL1 results in the malfunction of the PI3K complex, causing pleiotropic organ developmental defects by affecting the LE morphology, retromer function and PIN1 polar localization in the plant cells (Fig. 8).

In yeast, Vps38p was identified to be necessary to sort the vacuolar hydrolase carboxypeptidase Y (CPY) (Raymond *et al*., 1992). Vps38p forms a complex with PI3K in CPY targeting (Kihara *et al*., 2001). Recently, the crystal structure of the PI3K complex revealed that Vps38p interacts with Vps30p to form a parallel heterodimer that fits like a bracket around the Vps15p and Vps34p heterodimer (Rostislavleva *et al*., 2015). Vps38p is highly conserved in eukaryotes (Itakura *et al*., 2008; Sun *et al*., 2008; Matsunaga *et al*., 2009). In mammals, the Vps38p ortholog UVRAG was initially identified by partially complementing the UV sensitivity of xeroderma pigmentosum cells (Perelman *et al*., 1997), and UVRAG participates in the development of a variety of human malignancies including breast and colon cancer (Bekri *et al*., 1997; Goi *et al*., 2003; Liang *et al*., 2007; He & Liang, 2015). UVARG also forms a PI3K complex containing VPS34, Beclin1/VPS30 and VPS15 in mammals (Liang *et al*., 2006; Takahashi *et al*., 2007; Itakura *et al*., 2008; Matsunaga *et al*., 2009; Zhong *et al*., 2009). In *Arabidopsis*, we previously found that AtVPS15 and AtVPS30 are essential for the development of male gametophytes (Qin *et al*., 2007; Wang *et al*., 2012). No homozygous mutants of *atvps15* or *atvps30* could be obtained in the progeny of the heterozygous +/*atvps15* or +/*atvps30* (Fujiki *et al*., 2007; Qin *et al*., 2007; Xu *et al*., 2011; Wang *et al*., 2012). The disruption of AtVPS34 also causes male gametophytes to be lethal (Lee *et al*., 2008), indicating that the PI3K complex is pivotal for plant reproduction. In this study, we identified that USL1 was the possible ortholog of Vps38/UVRAG in *Arabidopsis*. USL1 contains a CC1 and a CC2 domain in the N‐terminus and a BARA domain in the C‐terminus similar to those in Vps38/UVRAG. We also demonstrate that USL1 forms a complex with AtVPS15, AtVPS30 and AtVPS34. USL1 directly interacts with AtVPS30 using the CC1 and CC2 domains. These data suggest that the function of Vps38p/UVRAG/USL1 is conserved not only in yeast and animals, but also in plants. However, unlike *AtVPS15*,* AtVPS30* or *AtVPS34*, the disruption of *USL1* does not cause the gametophytes to be lethal. We obtained homozygous *usl1* mutants, and they displayed severe vegetative phenotypes including the production of small and curled leaves. These results imply that some additional proteins could play redundant or independent functions in the regulation of PI3K during plant pollen development, because PI3K can also form an independent complex with Atg14 in addition to that with Vps38/UVRAG in yeast and animals (Kihara *et al*., 2001; Itakura *et al*., 2008). Alternatively, the roles of *USL1* in leaf and other organ development implied that *AtVPS15*,* AtVPS30* or *AtVPS34* could also not only be essential for gametophyte development, but also for vegetative organ development in plants.

Membrane trafficking plays an important role in plant development and growth. During endocytosis, the membrane proteins are internalized to the cytoplasm and then transported to the early endosomes (EE). The EE then mature to become the LE/PVC/MVB where the cargo proteins are either transported forward to the vacuoles for degradation or back to the EE for recycling (Fan *et al*., 2015). The Rab GTPases act as molecular switches for membrane trafficking by converting between GDP‐ and GTP‐bound states. RABF2a is a Rab GTPase whose GTP‐bound activated state is localized to the LE/PVC/MVB, whereas the GDP‐bound inactivated RABF2a is detached from the LE into the cytosol during GTPase cycling (Goh *et al*., 2007). Thus, RABF2a is used as a molecular marker labeling LE/PVC/MVB (Geldner *et al*., 2009). We show that USL1 is colocalized with RABF2a in the LE/PVC/MVB. The disruption of the *USL1* function causes an enlarged ring‐like LE/PVC/MVB, a structure similar to that in the plants treated with the PI3K inhibitor Wortmannin. This is consistent with our result that USL1 interacts with the PI3K complex and regulates PI3K function. Similar ring‐like LE/PVC/MVB structures were also observed in the plant cells in which the constitutively GTP‐bound mutant ARA7/RABF2b (Q69L) was overexpressed (Jia *et al*., 2013). Inactivation of the RAB GTPase requires the GTPase activating protein (GAP). Interestingly, TB2C that is a Rab GAP has recently been found to be recruited by VPS34 to inactivate RAB5 Rab GTPase during endosome maturation in *Caenorhabditis elegans* (Law *et al*., 2017). Although the exact mechanisms by which USL1 regulates the morphology of the LE/PVC/MVB remain unclear, we speculate that the loss of function of USL1 might cause the abnormal function of AtVPS34 to affect the recruitment of Rab GAPs to inactivate RABF2a/2b, leading to the ring‐like LE/PVC/MVB resulting from the constitutive activation of RABF2a/2b.

Auxin acts as a master regulator of plant development. The polar auxin transport mediated by the PIN efflux carriers is critical to form the auxin gradient essential for plant organ development (Grunewald & Friml, 2010). PIN1 is one of the most important PIN proteins that is asymmetrically localized to the cell PM, causing auxin flow direction in plant tissues. PIN1 proteins are recycled between the PM and the cytosolic membrane, and the polar localization of the PIN1 is regulated by membrane trafficking. During the past decades, several important components of endocytic trafficking have been identified to be important for PIN1 trafficking and localization during plant leaf and other organ development. In addition to the GNOM protein and ESCRT complex that is important for PIN1 trafficking (Geldner *et al*., 2003; Reyes *et al*., 2011; Gao *et al*., 2014), *VAN4* encoding a GEF for the RAB GTPase controls plant vascular development by mediating PIN1 trafficking (Naramoto *et al*., 2014a). Additional factors including BEX1/ARF1A/1C, BEX5/RabA1b, BEN1 and BEN2/VPS45 also participate in the membrane trafficking of PIN proteins (Tanaka *et al*., 2009, 2014; Feraru *et al*., 2012). VPS51 regulates leaf shape and vein patterning by targeting PIN1 to the lytic vacuole for degradation (Pahari *et al*., 2014), whereas VPS29 and SNX1 are the retromer components that are required for the retrieval of PIN1 from the LE/PVC/MVB back to the EE for recycling (Jaillais *et al*., 2006, 2007). Both of these are localized to the LE/PVC/MVB. The disruption of VPS29 causes severe auxin‐related plant organ development phenotypes by affecting PIN1 recycling, indicating that the retromer plays a central role in the regulation of PIN1 trafficking (Jaillais *et al*., 2006, 2007). In this study, we found that USL1 co‐localized with VPS29. The morphology of the VPS29‐labeled endosome was aberrant in the *usl1* mutants. The *usl1* mutants display developmental defects similar to those observed in the *vps29* mutant. The homozygous *vps29 usl1* double mutants are lethal, whereas the homozygous *vps29* or *usl1* mutants are essential, indicating that *USL1* acts in parallel with *VPS29*. We also found that *usl1* interacts synergistically with *pin1*. These data suggest that USL1 acts in the same pathway with VPS29 and PIN1 and plays a pivotal role in regulating the morphology of LE, the retromer function and PIN1 recycling. Interestingly, in yeast, the USL1 ortholog Vps38p was also found to regulate the retromer function (Burda *et al*., 2002). The Vps30p–Vps38p complex binds to Vps34p for the proper localization of the retromer Vps5p–Vps17p complex (Burda *et al*., 2002). In animals, the PI3K complex also controls the retromer function during plgR‐plgA transcytosis in epithelial cells (Vergés *et al*., 2007). These data provide further evidence that the function of USL1/Vps38p/UVRAG is highly conserved in yeasts, animals and plants.

In summary, we identified the important factor USL1 that plays pivotal roles in plant development by regulating the PI3K complex, retromer function and the maintenance of the LE/PVC/MVB morphology and auxin distribution. As sessile organisms, plants evolve morphological adaptations in response to different growth conditions. USL1 may control the polarity and abundance of the PIN1 proteins and other proteins that depend on retromer function and the normal morphology of LE, therefore affecting plant development in response to internal and external signals. It will be very interesting to identify how *USL1* is regulated by different developmental or environmental cues in the future.

## Author contributions

G.Q. conceived the project; G.Q. and R.Y. designed the experiments; R.Y., J.L., Y.F., H.Y., J.Z. and G.Q. performed the experiments; G.Q., R.Y. and J.H. analyzed the data; and G.Q. and R.Y. wrote the paper.

## Supporting information

Please note: Wiley Blackwell are not responsible for the content or functionality of any Supporting Information supplied by the authors. Any queries (other than missing material) should be directed to the *New Phytologist* Central Office.

**Fig. S1** The 50‐d‐old wild‐type and rescued *usl1‐1* with USL1pro‐USL1‐GFP in Arabidopsis.
**Fig. S2** Domain analysis of USL1 in Arabidopsis.
**Fig. S3** The other phenotypes of the Arabidopsis mutant *usl1‐2*.
**Table S1** The list of primers used in this study
**Table S2** The auxin‐related genes upregulated in the Arabidopsis mutant *usl1‐2*
**Table S3** The auxin‐related genes downregulated in the Arabidopsis mutant *usl1‐2*
**Table S4** The leaf flattening‐related genes regulated in the Arabidopsis mutant *usl1‐2*

**Notes S1** The genes upregulated in the Arabidopsis mutant *usl1‐2*.
**Notes S2** The genes downregulated in the Arabidopsis mutant *usl1‐2*.
**Notes S3** The gene ontology (GO) enrichment analysis of upregulated genes in the Arabidopsis mutant *usl1‐2*.
**Notes S4** The gene ontology (GO) enrichment analysis of downregulated genes in the Arabidopsis mutant *usl1‐2*.

**Movies S1** Colocalization of USL1‐GFP and RABF2a‐mCherry in Arabidopsis.

**Movies S2** Colocalization of USL1‐GFP and VPS29‐RFP in Arabidopsis.
