# Supplementary material for: The Arabidopsis USL1 controls multiple aspects of development by affecting late endosome morphology
Source: New Phytol. 2018 Jun 13;219(4):1388–405. doi: 10.1111/nph.15249 (PMC6099276; doi:10.1111/nph.15249)
Supplement: Supplementary file 1 — Fig. S1 The 50‐d‐old wild‐type and rescued usl1‐1 with USL1pro‐USL1‐GFP in Arabidopsis. Fig. S2 Domain analysis of USL1 in Arabidopsis. Fig. S3 The other phenotypes of the Arabidopsis mutant usl1‐2. Table S1 The list of primers used in this study Table S2 The auxin‐related genes upregulated in the Arabidopsis mutant usl1‐2 Table S3 The auxin‐related genes downregulated in the Arabidopsis mutant usl1‐2 Table S4 The leaf flattening‐related genes regulated in the Arabidopsis mutant usl1‐2 [file NPH-219-1388-s001.pdf]

New Phytologist **Supporting Information**

Article title: **The *Arabidopsis* USL1 Controls Multiple Aspects of Development by Affecting Late Endosome Morphology**

Authors: Rongrong Yuan[1, 2], Jingqiu Lan[1], Yuxing Fang[1], Hao Yu[1], Jinzhe Zhang[1], Jiaying Huang[1], Genji Qin[1, *]

Article acceptance date: *21 April 2018*

The following Supporting Information is available for this article:

Fig. S1 The 50-d-old wild-type and rescued *usl1-1* with USL1pro-USL1-GFP in Arabidopsis.

Fig. S2 Domain analysis of USL1 in Arabidopsis.

Fig. S3 The other phenotypes of the Arabidopsis mutant *usl1-2*.

Table S1 The primers used in this study.

Table S2 The auxin-related genes up-regulated in the Arabidopsis mutant *usl1-2*.

Table S3 The auxin-related genes down-regulated in the Arabidopsis mutant *usl1-2*.

Table S4 The leaf flattening-related genes regulated in the Arabidopsis mutant *usl1-2*.

Movie S1 Colocalization of USL1-GFP and RABF2a-mCherry in Arabidopsis.

Movie S2 Colocalization of USL1-GFP and VPS29-RFP in Arabidopsis.

Fig. S1 The 50-d-old wild type and rescued *usl1-1* with USL1pro-USL1-GFP in Arabidopsis. Bar = 1 cm.

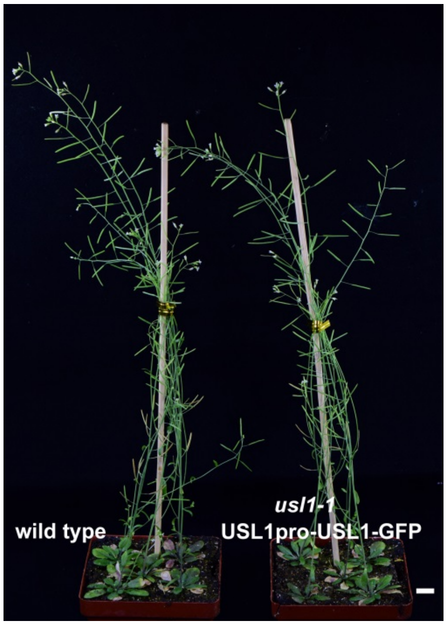

Fig. S2 Domain analysis of USL1 in Arabidopsis. (a) The second structure of USL1 was predicated on the structure of yeast Vps38p. USL1-P, predicted secondary structure; USL1-S, USL1 sequence; Vps38p-S, Vps38p template sequence; Vps38p-K, Vps38p known secondary structure; Vps38p-P, Vps38p predicted secondary structure. (b) The predicted structure of USL1. (c) The structure of Vps38p.

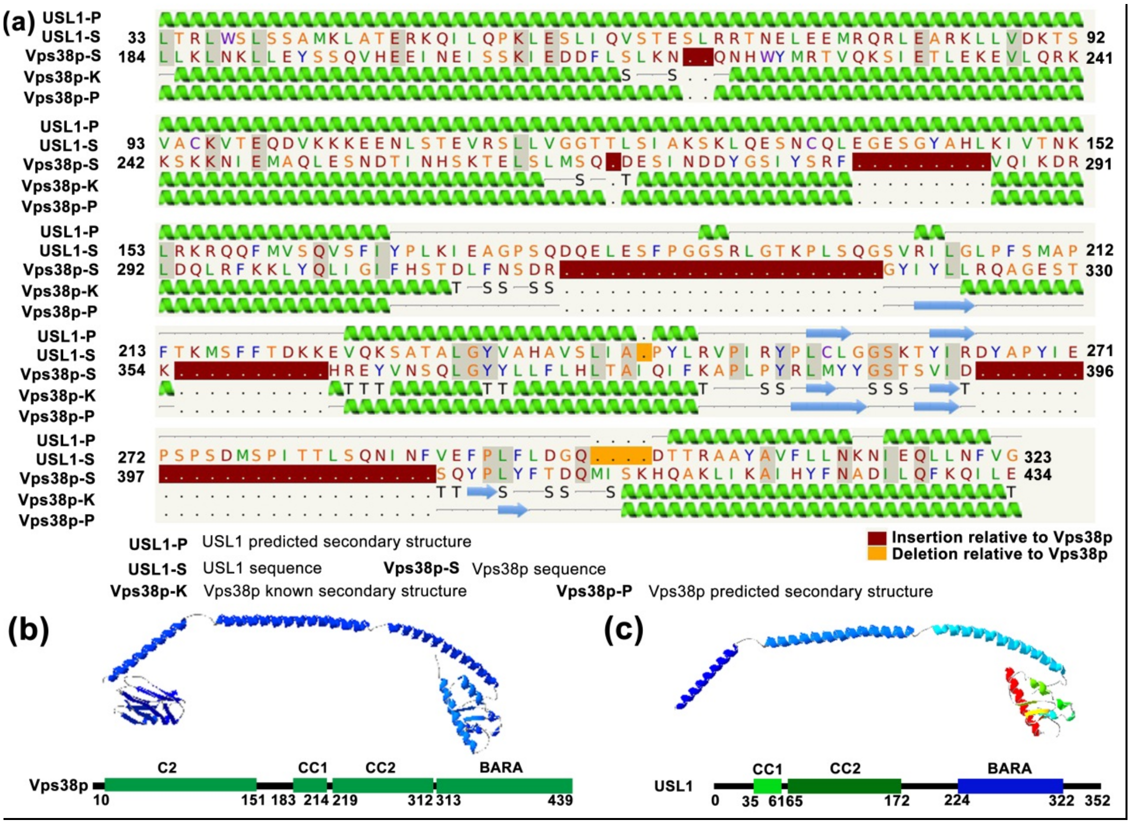

Fig. S3 The other phenotypes of the Arabidopsis mutant *usl1-2*. (a) 40-d-old wild type control and *usl1-2*. *usl1-2* displayed curly leaves and late flowering. (b) 60-d-old wild type control and *usl1-2*. *usl1-2* displayed low fertility and abnormal silique phyllotaxy. Bar = 1 cm.

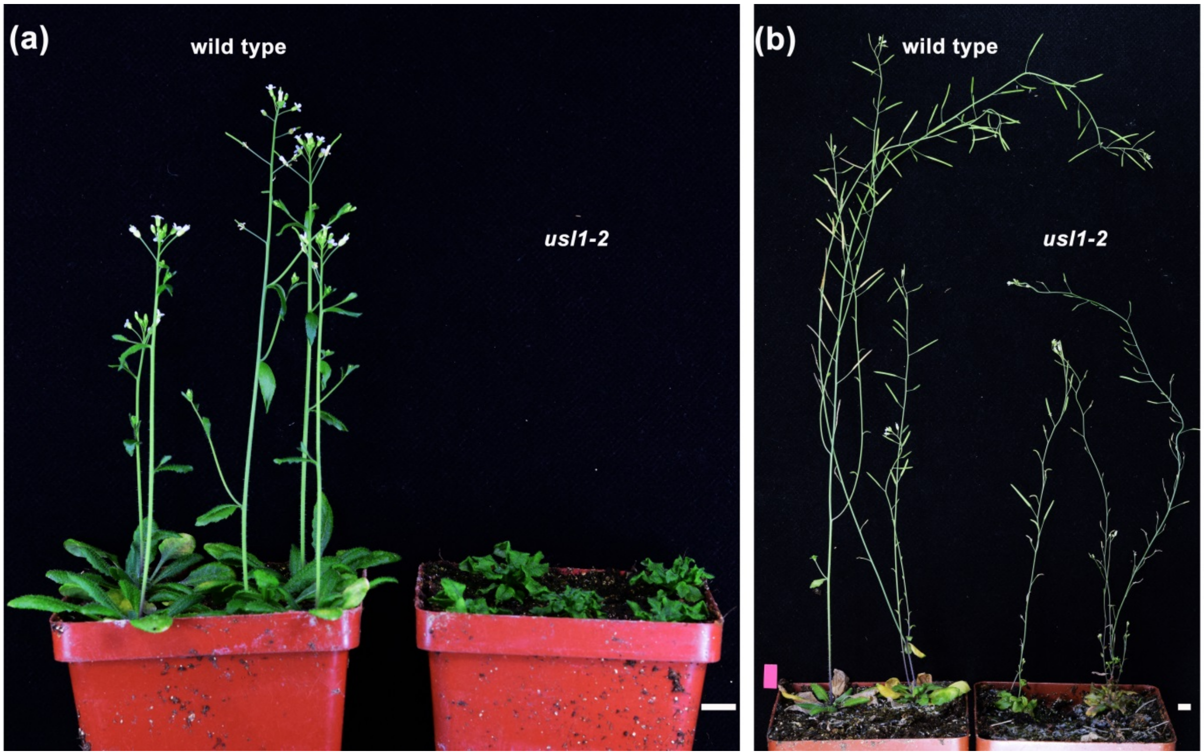

Table S1 The primers used in this study.

Primer name	Primer sequences(5'-3')
**Primers for genotyping T-DNA insertion mutants**	
usl1-1-LP	TAAGCTTTCGTTTATCGTCGG
usl1-1-RP	GCCCATGTATGTTCCGTCCT
usl1-2-LP	CTAATCCGATCTCTCGGTTCC
usl1-2-RP	TCTCCTTCTAGTTGGCAGTTTG
usl1-2-TRP	GGGCAGGTTAGTACTGAATCATTG
pin1-LP	GGTGATGCCACCAACAAGTG
pin1-RP	CATCACAGCCACTGATCCACC
LB3	TAGCATCTGAATTTCATAACCAATCTCGATACAC
LBb1.3	ATTTTGCCGATTTCGGAAC
**Primers for cloning**	
VPS29F	CACCATGGTGCTGGTATTGGCATTG
VPS29R	CTACGGACCAGAGCTGGTAG
VPS29Rnsc	CGGACCAGAGCTGGTAGTAG
VPS30F	CACCATGAGGAAAGAGGAGATTCC
VPS30R	CTAAGTTTTTTTACATGAAGGC
VPS30Rnsc	AGTTTTTTTACATGAAGGC
VPS34F	CACCATGGGTGCGAACGAGTTTCG
VPS34R	TCAACGCCAGTATTGAGCCCAT
USL1F	CACCATGGAAAGAGTATCAGAGCG
USL1proF	CACCGCCTTAAGACATTGTTAATG
USL1proR	CAACAATTATCTCGGAACCC
USL1Rnsc	TAAATAATCGATATAAT
USL1R	TCATAAATAATCGATATAAT
USL1△NF	CACCATGGAAGTTCAGAAGTCTGCAAC
USL1△CR	TTTCTTGTCGGTGAAGAAGCTC
USL1△CC1F	TGAGCTTACGCAGGTTAGTACTGAATC
USL1△CC1R	GTACTAACCTGCGTAAGCTCATGGTCG
USL1△CC2F	TCAGGTTAGTGAAGCTGGACCTTCACAAG
USL1△CC2R	GGTCCAGCTTCACTAACCTGAATAAG
USL1△CC1genomR	TAAGAGAAAAACCTGCGTAAGCTCATGGTCGAATTCTTC
USL1△CC1genomF	GACCATGAGCTTACGCAGGTTTTTCTCTTACTCATG
USL1△CC2genomR	TGAAGGTCCAGCTTCACTAACCTGCCCGAAATTTTG
USL1△CC2genomF	TTCGGGCAGGTTAGTGAAGCTGGACCTTCACAAGAC
**Primers for testing expression level of genes**	
SALK_094540 RT F1	CTATGCACCTTACATTGAGCC
SALK_094540 RT R1	CAAAGTTCAAGAGTTGCTCG
SAIL_552_F02 RT F2	GAGAGGAAACAAATCCTGC
SAIL_552_F02 RT R2	CCGCCTACTAGCAAAGACC

Table S2 The auxin-related genes up-regulated in the Arabidopsis mutant *usl1-2*.

Gene_ID	Gene Product	WT_FPKM	*usl1*_FPKM	Log2(fold_change)	P_Value
AT3G53250	SAUR57	0.58	4.06	2.8	0.0284
AT5G18050	SAUR22	0.47	2.60	2.4	0.0086
AT5G18010	SAUR19	0.67	3.65	2.4	0.00505
AT3G44300	NIT2	16.10	76.46	2.3	5.00E-05
AT3G03830	SAUR28	0.77	3.2	2.1	0.01065
AT4G38825	SAUR13	0.49	1.98	2.0	0.00855
AT5G18060	SAUR23	0.95	3.705	2.0	0.00415
AT1G29460	SAUR65	2.76	10.15	1.9	5.00E-05
AT1G29450	SAUR64	3.89	12.44	1.7	5.00E-05
AT5G50760	SAUR55	17.73	50.46	1.5	5.00E-05
AT3G03840	SAUR27	1.21	3.37	1.5	0.0004
AT5G18080	SAUR24	3.44	9.36	1.4	0.00385
AT1G56010	NAC1	6.87	17.75	1.4	5.00E-05
AT5G18030	SAUR21	4.44	11.454	1.4	5.00E-05
AT3G03850	SAUR26	1.74	4.40	1.3	0.00045
AT4G34790	SAUR3	0.58	1.43	1.3	0.04455
AT5G18020	SAUR20	5.53	11.63	1.1	5.00E-05
AT5G65510	PLT7	0.89	1.82	1.0	0.00075

Table S3 The auxin-related genes down-regulated in the Arabidopsis mutant *usl1-2*.

Gene_ID	Gene Product	WT_FPKM	*usl1*_FPKM	Log2(fold_change)	P_Value
AT4G22620	SAUR34	3.39	0.37	-3.2	0.0041
AT2G35290	SAUR79	6.59	1.02	-2.7	5.00E-05
AT2G21200	SAUR7	15.33	2.62	-2.5	5.00E-05
AT1G05680	UGT74E2	49.57	10.68	-2.2	5.00E-05
AT3G62150	PGP21	9.99	3.18	-1.6	5.00E-05
AT5G16530	PIN5	4.65	1.69	-1.5	5.00E-05
AT5G54490	PINOID-BINDING PROTEIN 1	4.82	1.76	-1.5	0.001
AT4G34810	SAUR5	2.43	0.97	-1.3	0.05355
AT3G59900	ARGOS	26.49	11.15	-1.2	5.00E-05
AT5G43890	YUC5	2.61	1.10	-1.2	5.00E-05
AT4G34800	SAUR4	3.87	1.69	-1.2	0.14565
AT5G10990	SAUR69	4.67	2.18	-1.1	0.0001
AT5G57420	IAA33	1.34	0.67	-1.0	0.1206

Table S4 The leaf flattening-related genes regulated in the Arabidopsis mutant *usl1-2*.

Gene_ID	Gene Product	WT_FPKM	*usl1*_FPKM	Log2(fold _change)	P_Value
AT3G18010	WOX1	6.90	3.20	-1.1	5.00E-05
AT2G28610	PRS/WOX3	0.39	0.17	-1.2	1.0000
AT5G08070	TCP17	4.32	2.25	-0.9	0.0009
AT4g28840	TIE1	1.46	2.52	0.8	0.0112
AT2g34010	TIE4	0.51	0.84	0.7	0.0536

Notes S1 The genes up-regulated in the Arabidopsis mutant *usl1-2*.

Notes S2 The genes down-regulated in the Arabidopsis mutant *usl1-2*.

Notes S3 The Gene Ontology (GO) enrichment analysis of up-regulated genes in the Arabidopsis mutant *usl1-2*. The items mentioned in the main text were marked in yellow shading.

Notes S4 The Gene Ontology (GO) enrichment analysis of down-regulated genes in the Arabidopsis mutant *usl1-2*. The items mentioned in the main text were marked in yellow shading.

Movie S1 Colocalization of USL1-GFP and RABF2a-mCherry in Arabidopsis. The left was the USL1-GFP localization, the middle was the RABF2a-mCherry localization and the right was the merged one. The timeline was shown above the picture. Bar = 60 μm.

Movie S2 Colocalization of USL1-GFP and VPS29-RFP in Arabidopsis. The left was the USL1-GFP localization, the middle was the VPS29-RFP localization, and the right was the merged one, and the timeline was shown above the picture. Bar = 60 μm.
